# Randomized clinical trial of an Acceptance and Commitment Therapy telehealth intervention (‘*REVITALIZE*’) to reduce fatigue interference in women with advanced ovarian cancer on PARP inhibitors: study protocol

**DOI:** 10.1186/s12885-026-15936-0

**Published:** 2026-04-02

**Authors:** Joanna J. Arch, Hajime Uno, Hanneke Poort, Elizabeth Slivjak, Madeline S. Nealis, Hannah Park, Irene Wang, Sarah R. Genung, Angel Cronin, David J. Andorsky, Laura J. Havrilesky, Richard T. Penson, Varvara Mazina, Page Widick, Carolyn Lefkowits, Alexi A. Wright

**Affiliations:** 1 Department of Psychology and Neuroscience, University of Colorado Boulder, Boulder, CO USA; 2 Division of Cancer Prevention and Control, University of Colorado Cancer Center, Aurora, CO USA; 3 Department of Medical Oncology, Division of Population Sciences, and Harvard Medical School, Dana-Farber Cancer Institute, Boston, MA USA; 4 Radboud University Medical Center, Nijmegen, Netherlands; 5 Rocky Mountain Cancer Centers, Boulder, CO USA; 6 Division of Gynecologic Oncology, Department of Obstetrics and Gynecology, Duke Cancer Institute, Duke School of Medicine, Durham, NC USA; 7 Division of Medical Oncology, Harvard Medical School, Massachusetts General Hospital, Boston, MA USA; 8 Division of Medical Oncology, Department of Medicine, Harvard Medical School, Beth Israel Deaconess Medical Center, Boston, MA USA; 9 Division of Gynecologic Oncology, University of Colorado Anschutz School of Medicine, Aurora, CO USA; 10 Department of Medical Oncology, Division of Gynecologic Oncology, Dana-Farber Cancer Institute, Boston, MA USA

**Keywords:** Ovarian cancer, Cancer, Fatigue, Quality of life, Medication adherence, Acceptance and commitment therapy, PARP inhibitors, Maintenance treatment

## Abstract

**Background:**

In the era of precision oncology, patients with advanced cancer are often living longer but are enduring years of fatiguing treatment. Fatigue is among the most common, chronic, and under-addressed side effects of advanced cancer treatment, and can lead to dose reductions, treatment interruptions, and discontinuation. Oral PolyADP-ribose polymerase (PARP) inhibitors have revolutionized the treatment of advanced ovarian cancer but are often accompanied by clinically-significant fatigue, and evidence-based treatments to cope with this side effect remain limited. A pilot trial suggests that telehealth acceptance and commitment therapy (ACT), a variant of cognitive behavioral therapy based on acceptance, mindfulness, and personal values, has potential to improve fatigue interference (i.e. disrupting activity and quality of life) among ovarian cancer patients on maintenance PARP inhibitors. This study protocol describes a fully-powered randomized controlled trial (RCT) of an innovative, remotely-delivered ACT intervention (‘*REVITALIZE*’) that aims to improve fatigue outcomes and PARP inhibitor adherence.

**Methods:**

This two-armed, prospective RCT randomizes 240 adults with advanced ovarian cancer who report moderate to severe fatigue while on PARP inhibitors 1:1 to either *REVITALIZE* or education-enhanced usual care. The *REVITALIZE* intervention includes eight weekly one-hour individual sessions delivered via videoconference, plus two booster sessions at one- and two-month intervals thereafter. Participants, recruited from both academic and community cancer care sites across three U.S. regions, will complete assessments at baseline, mid-intervention, post-intervention, and 20 and 28-week follow-up (FU). The primary patient-reported outcome is change in fatigue interference from baseline to week 20 (FU1). Secondary patient-reported outcomes include fatigue severity, catastrophizing, self-efficacy, and quality of life. The primary behavioral outcome is monthly PARP inhibitor adherence, assessed by wireless medication adherence monitors; dose interruptions, reductions, and persistence will also be evaluated using adherence monitors and medical chart review.

**Discussion:**

This paper describes a RCT that will evaluate the efficacy of the telehealth *REVITALIZE* intervention for improving fatigue-related outcomes and medication adherence among adults with advanced ovarian cancer taking PARP inhibitors. Findings will inform clinical practice and supportive care for adults with advanced ovarian cancer receiving treatments that cause fatigue.

**Trial registration:**

Clinicaltrials.gov NCT06710548 on November 29, 2024.

**Supplementary Information:**

The online version contains supplementary material available at 10.1186/s12885-026-15936-0.

## Background

Fatigue is one of the most common side effects experienced by patients with advanced cancer and is a pressing problem [1–3] that remains vastly underreported, underdiagnosed, and undertreated [4, 5]. In the era of precision oncology, novel treatments such as targeted therapies, anti-angiogenic agents, immunotherapy, and antibody-drug conjugates [2, 3] are extending survival [6–8], yet creating a growing population of advanced cancer patients who remain on treatment for years and experience clinically significant, enduring fatigue as a result. Unaddressed fatigue can compromise cancer treatment outcomes as a dose-limiting side effect or by reducing adherence to oral therapies [9]. Unlike other common treatment-related side effects (e.g., nausea, pain), pharmacologic strategies are largely ineffective at addressing fatigue [10–12]. For patients requiring ongoing maintenance treatment, it is more realistic to reduce the *interference* of fatigue on daily life (‘fatigue interference’) than eliminate fatigue altogether. However, apart from a recent breast cancer trial [13], few studies in advanced cancer have examined this outcome.

Ovarian cancer offers an ideal context to evaluate interventions to reduce fatigue interference in patients with advanced cancer. Nearly 80% of patients with ovarian cancer are diagnosed with advanced disease, stage III or IV, which is typically incurable [14]. Most patients receive 2–3 years of oral poly ADP-ribose polymerase (PARP) inhibitor treatment [15–17], which has led to an unprecedented 70% reduction in the 7-year risk of cancer recurrence or death in patients with *BRCA-*associated advanced ovarian cancer compared to placebo [18] and improvements in progression-free and overall survival in patients with homologous-recombination-deficient ovarian cancers [6], reflecting a major success in precision oncology.

However, the effectiveness of PARP inhibitors, like many breakthrough treatments, comes at a cost: more than 40% of patients taking PARP inhibitors experience significant enduring fatigue. A recent meta-analysis indicated that PARP inhibitors are associated with a 2.23 relative risk of high-grade (more severe) fatigue compared with placebo [19]. In our qualitative study, patients described PARP inhibitor-associated fatigue as unrelenting and pervasive, interfering with work, family, and social events, and eroding their sense of self [20]. In contrast to fatigue caused by time-limited chemotherapy or radiation, patients with advanced ovarian cancer must take oral PARP inhibitors once or twice daily (depending on regimen) for years. Because PARP inhibitors were first FDA approved in 2014, their fatigue effects are better characterized than more emerging treatments, establishing a strong scientific foundation for addressing them.

Meta-analytic evidence to date shows that after primary cancer treatment, cognitive behavioral therapy (CBT) is more effective for reducing cancer-related fatigue than other interventions (pharmacologic, exercise, other psychological) [12]. Patients with advanced cancers more frequently experience enduring fatigue than those with early-stage cancers [9]—yet the same meta-analysis found that fewer than 10% of studies have tested interventions for patients with advanced disease [12]. Further, improvements in fatigue were significantly smaller for patients with advanced disease compared to those with early-stage disease. A separate Cochrane systematic review and meta-analysis of fatigue interventions for patients with advanced cancer (most included early-stage survivors but they examined the subgroup with advanced cancer) [4] demonstrated significant reductions in fatigue only at first follow-up, but not post-intervention or the second follow-up, indicating a response that was not sustained. In addition, fatigue *interference* was not examined. Overall, the evidence was rated “very low quality” and likely biased due to small sample sizes (typically fewer than 50 advanced cancer patients per study arm).

The only known published fully powered randomized trial of an intervention to address fatigue in patients with advanced cancer used a variant of CBT to address fatigue interference in patients with metastatic breast cancer [21]. Although this trial makes an important contribution, it focused exclusively on breast cancer, the area in which most fatigue research has been conducted [12], and did not focus on fatigue interference caused by novel treatments such as PARP inhibitors. Finally, to our knowledge, very few studies have investigated whether addressing fatigue boosts cancer treatment adherence – a critically important question to advance both science and clinical practice. The current trial addresses these important gaps in a well-powered, high-quality CBT trial that addresses fatigue or its interference in the lives of patients with advanced ovarian cancer.

### The current intervention approach and pilot findings

This trial evaluates *REVITALIZE*, an intervention our team developed based on Acceptance and Commitment Therapy (ACT) [22, 23]. ACT is a variant of CBT that combines acceptance, mindfulness, and behavior-change strategies to help people cope – especially with problems that may not resolve [24, 25]. We systematically adapted ACT to address fatigue and fatigue interference challenges by first conducting a qualitative interview study among patients with advanced ovarian cancer on PARP inhibitors (*n* = 23) to understand fatigue’s impacts on daily life [20]. Participants described profound, life-limiting fatigue. For example, one participant said, “I worked at my job for over 30 years.outside, running around…and now I’m only working two hours a day…” (p. 230). Due to PARP inhibitor-related fatigue, several patients had to retire early. Another said, “You feel like ‘God, I’m so tired of being tired.’ And you don’t know where the end is…because it’s not really in sight. And it’s like ‘Oh God, I’m going to feel like this the rest of my life?’” (p. 231). Based on these findings, we developed *REVITALIZE*, an ACT-based 1:1 (individually delivered) intervention to address fatigue and fatigue interference caused by ongoing advanced cancer treatment.

In contrast to mastery and control-based behavioral interventions designed to minimize or eliminate symptoms [26], *REVITALIZE* adapts ACT to help advanced cancer patients cope with the enduring challenge of remaining on fatiguing treatment by reducing fatigue interference on their daily quality of life [24]. *REVITALIZE* helps patients with advanced cancer shift how they experience and cope with ongoing fatigue by actively accepting their experience of fatigue rather than spending precious energy trying to control, eliminate, or avoid it, by regulating their sleep rhythms to prevent fatigue worsening, and by reinvesting their limited energy to pursue, in a balanced way, what brings them vitality and meaning.

We conducted a pilot randomized trial of a weekly 6-session *REVITALIZE* intervention (with a 1-month booster session) relative to an education control (*n* = 44). Findings demonstrated strong feasibility, acceptability, and significant reductions in fatigue interference, severity, and catastrophizing, as well as improvements in quality of life and PARP inhibitor adherence following *REVITALIZE* relative to the education control condition [27]. In exit interviews, both *REVITALIZE* participants and study interventionists reported a desire to extend the intervention by several sessions to improve pacing, increase practice opportunities, and add another booster session. Thus, the current trial will test an 8-session version of *REVITALIZE* (plus booster sessions 1 and 2 months later) relative to an education control condition.

For accessibility, particularly in rural areas, and to reduce transportation burden on participants, *REVITALIZE* is designed for delivery via online videoconferencing. In the pilot trial, we developed and refined visual slides for each *REVITALIZE* session and a patient workbook that illustrates key intervention concepts and skills for both in-session and at-home practice.

### The current study protocol

This protocol describes a two-arm, prospective randomized controlled trial (RCT) to evaluate the efficacy of the *REVITALIZE* intervention in reducing fatigue interference and improving adherence to PARP inhibitors, compared with an education-enhanced usual care condition (EUC). Participants will be patients with advanced ovarian cancer who report moderate to severe fatigue (score of ≥ 4 on the Fatigue Symptom Inventory-3 screen [28, 29]) while on PARP inhibitors. This trial addresses four limitations of prior cancer-related fatigue research by: (1) focusing exclusively on patients with advanced cancer; (2) targeting fatigue associated with a novel precision oncology treatment (PARP inhibitors); (3) primarily targeting fatigue interference, a more realistic main target than fatigue severity among patients with advanced cancer requiring ongoing treatment that causes fatigue; (4) directly testing whether addressing fatigue improves cancer treatment adherence – an underexplored relationship that could inform clinical care.

Comparing *REVITALIZE* to an EUC condition helps to determine if *REVITALIZE* offers benefits beyond those realized by offering standardized cancer survivorship materials developed by the National Cancer Institute [30]. The aims of this study include:

1. Test the hypothesis that *REVITALIZE*, relative to EUC, will reduce the primary outcome of fatigue interference and reduce fatigue severity and catastrophizing and increase fatigue self-efficacy and quality of life (secondary outcomes).
2. Assess whether *REVITALIZE*, relative to EUC, improves monthly PARP inhibitor adherence, dose interruptions, reductions, and persistence, using wireless medication adherence monitors and medical chart review.
3. Test *REVITALIZE* theory-driven mechanisms of action; specifically, to see if *REVITALIZE* increases acceptance and alignment with values more than EUC, and whether changes in these putative mechanisms predict fatigue-related and quality of life outcomes at follow-up.

## Methods/design

### Study design and funding

This study is a two-arm, prospective RCT that aims to randomize 240 patients with advanced ovarian cancer who are currently prescribed PARP inhibitors and report moderate to severe fatigue. Participants are individually randomized 1:1 to either *REVITALIZE* or EUC based on a randomization block sequence generated by the study biostatistician (H.U.) using R statistical software [31], stratified by PARP inhibitor type and regimen (once versus twice daily and with versus without bevacizumab) and recruitment site. Patient-reported outcomes will be collected in Research Electronic Data Capture (REDCap) [32], a HIPAA-approved, secure, web-based tool, at five timepoints: baseline (pre-intervention), mid-intervention (8 weeks after baseline), post-intervention (13 weeks after baseline), and 20- and 28-week follow-ups (FU). The 20-week FU serves as the main endpoint, and the 28-week FU serves to assess maintenance or change in effects over time. Adherence outcomes will be assessed via Wisepill, a wireless adherence tracking device [33] and medical chart review.

The study was pre-registered with identifier NCT06710548 on Clinicaltrials.gov on November 29, 2024, before enrolling the first patient (https://clinicaltrials.gov/study/NCT06710548). Dana-Farber Cancer Institute serves as the single IRB overseeing the study and informed consent is obtained from all individuals prior to study enrollment.

### Study eligibility criteria

#### Inclusion criteria

Adult (age ≥18 years), English-speaking patients with advanced ovarian cancer will be eligible to participate if they:

1. Have epithelial ovarian cancer (including ovarian, fallopian tube, and primary peritoneal cancers, all of which are treated as ovarian cancers).
2. Are being treated with PARP inhibitors as a maintenance therapy after completion of first-line treatment or platinum-sensitive recurrent disease, and have taken the PARP inhibitors for at least 2 months. This corresponds to the time period when patients frequently undergo dose reductions, delays due to symptoms and side effects, and switches to other PARP inhibitors. After 2 months, most patients are on a stable dose that can be continued for the duration of their treatment.
3. Anticipate continued treatment on a PARP inhibitor for at least 8 months at time of consent (e.g., beyond the duration of the study).
4. Report an average rating of ≥ 4 (range 0–10) on the first 3 items of the Fatigue Symptom Inventory [28, 29], which rates fatigue levels during the past week on the most and least fatiguing day and on an average day. We used this same cutoff score in the REVITALIZE pilot study [27].
5. Report a rating of 0 to 2 on the Eastern Cooperative Oncology Group Performance Status Scale (ECOG) performance status at time of consent [34], indicating that they are out of bed most of the day.
6. Are capable at time of consent of understanding and voluntarily consenting to the study, attending online sessions, and completing homework for the intervention, which includes engaging in valued activities. Also, are willing to use a wireless electronic pillbox for PARP inhibitor medication.
7. Complete the baseline (pre-intervention) survey.

#### Exclusion criteria

1. Have an untreated clinical condition (e.g., anemia or hypothyroidism) or comorbid condition (e.g., chronic fatigue syndrome) that pre-dates PARP inhibitor use and could explain fatigue.
2. Have a history of chronic untreated trauma (unrelated to their cancer), psychiatric hospitalization or suicide attempt(s) in the past 5 years, or are currently at high risk for suicidality. Adults excluded for any of these reasons will be referred to broader or more intensive supportive resources.
3. Patients with cognitive conditions (e.g., dementia) determined by their treating oncologist or Site PI, such that they could not provide informed consent or complete the study procedures.

### Participant recruitment and consent

As ovarian cancer is a relatively rare form of cancer, participants will be recruited from multiple sites, including the Dana-Farber Cancer Institute (DFCI), Massachusetts General Cancer Center, and the Cancer Center at Beth Israel Deaconess Medical Center (BIDMC) in Massachusetts; University of Colorado Cancer Center in Colorado; and Duke Cancer Center in North Carolina. In addition, to better understand the intervention’s efficacy in real-world care settings, we will also recruit from the Rocky Mountain Cancer Centers (RMCC), the largest community provider of cancer care in Colorado, and DFCI-affiliated regional community oncology practices.

The academic cancer centers, which use the same electronic health record system (e.g., EPIC), will use methods from the pilot study [27] in which they automate identification of patients with advanced ovarian cancer and prescriptions for PARP inhibitors by creating an EPIC workbench or, alternately, obtaining a list of patients treated with PARP inhibitors from the cancer centers’ specialty pharmacists. Trained research assistants (RAs) will review the site’s workbench regularly and Site PIs at Massachusetts General Cancer Center and the Cancer Center at BIDMC will regularly review lists with pharmacists. After receiving permission from patients’ oncologists to approach potentially eligible patients, RAs will contact the patient in person, by telephone, or by secure email to describe the study. If patients are interested, the RA will administer the study eligibility screening. If the patient is eligible and interested, the RA will initiate an oral informed consent process using REDCap’s e-consent framework. After completing informed consent, participants will receive a copy of their completed consent form, asked to complete a baseline survey, and sent a wireless electronic pillbox. If recruitment rates are low at any site, study staff will undergo retraining and approach patients in person – a strategy successfully used during the pilot study.

At the primary community site of RMCC, we will use the triple strategy of practitioner referrals and outreach to potentially eligible patients, direct patient mailings, and onsite flyers—approaches we have used successfully in previous trials with RMCC [25, 35–38]. If needed, we will send an RA onsite to a large RMCC clinic to recruit, a strategy we have piloted but only use as a back-up strategy due to its time and resource demands. Once a potential participant contacts the study team for more information or is referred by a provider, the University of Colorado research team will contact them to share study details and, if interested, complete study eligibility screening. If the person is eligible and interested, the study’s oral informed consent process is initiated with consent documented using REDCap’s e-consent framework. After completion, participants are sent a copy, asked to complete a baseline survey, and provided with a wireless pillbox.

The consent form includes that study participation is entirely voluntary, that participants’ clinical care will not be compromised in any way if they withdraw from the study, and that participants can be removed at the discretion of the principal investigators (A.A.W. and J.J.A.) for reasons such as violating reasonable expectations for study participation.

### Study conditions

#### *REVITALIZE* content and flow

As presented in Table 1, the *REVITALIZE* intervention consists of eight weekly telehealth sessions followed by two telehealth booster sessions at one- and two-month follow-up (e.g., one and two months after the last weekly session). For replicability, each session is guided by a detailed interventionist manual and patient workbook. Weekly *REVITALIZE* sessions focus on patient acquisition of ACT skills and behavior changes tailored to address fatigue in advanced cancer. Specifically, the intervention uses metaphors, structured reflections, and experiential exercises to help patients: (1) acknowledge what they can versus cannot control about fatigue and cancer; (2) mindfully experience and accept fatigue as it shows up here-and-now, which is explored as a less draining alternative to spending significant energy fighting to get rid of it; (3) develop skills for responding to difficult thoughts, beliefs, and images about cancer and fatigue in ways that reduce their influence on well-being and daily behavior; (4) clarify and connect to personal values (what is most important to them); (5) sensitively guide participants to engage in values-based activities and set goals around such activities in a manner appropriate to their energy level; (6) regulate sleep/wake cycles to improve nighttime sleep, including reducing daytime sleep and sleep habits that interfere with nighttime sleep. Booster sessions focus on consolidating and troubleshooting skills and behavior changes to promote enduring benefit. As noted, in response to pilot study participant and interventionist feedback, this content is now covered over eight weekly sessions and two boosters rather than six weekly sessions and one booster (as in the pilot study [27]). However, intervention content remains the same.

**Table 1:** Schedule of REVITALIZE study enrollment, intervention, and assessment

	**Pre-randomization (Enrollment)**			**Post-randomization**						
	**Baseline**	**Device Mailing +Set-Up**	**PARPi Device Adherence Data Baseline**	**Intervention Start**	**Mid-point**	**Post- intervention**	**Booster 1**	**Booster 2**	**Primary Endpoint**	**Maintenance Follow-up**
	**Week 0**	**Week 1**	**Weeks 2-3**	**Week 4**	**Week 8**	**Week 13**	**Week 16 **	**Week 20**	**Week 20**	**Week 28**
ENROLLMENT:										
* 1. Eligibility screen*	**X**									
* 2. Informed consent*	**X**									
RANDOMIZATION				**X**						
REVITALIZE (one arm)				**X**	**X**	**X**	**X**	**X**		
ASSESSMENTS (both arms):										
* 1. Patient-Reported Outcomes*	**X**				**X**	**X**			**X**	**X**
* 2. Wisepill device data collection*			**X**	**X**	**X**	**X**	**X**	**X**	**X**	**X**

^**^Timepoints allow ±4 weeks

**Fig. 1:**
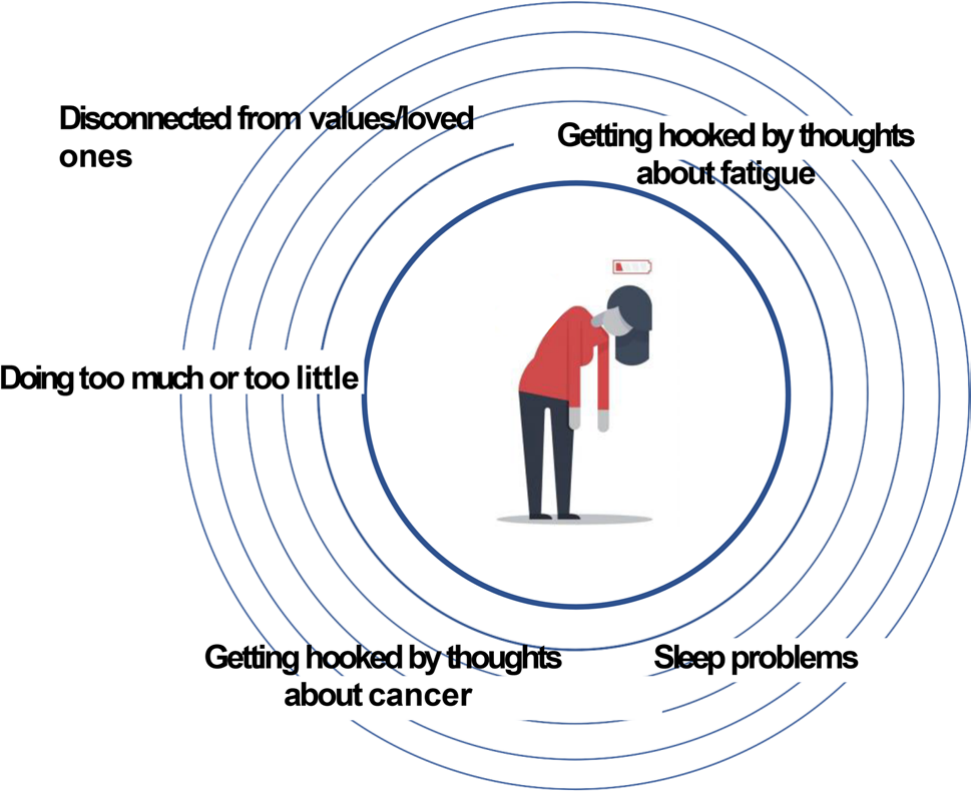
Sample REVITALIZE patient-facing slide

As in the pilot trial, the *REVITALIZE* intervention will be conducted by secure videoconferencing. Key concepts are communicated using engaging PowerPoint images (see Fig. 1). Parallel images and accompanying home practice worksheets are printed in patient workbooks that are mailed to participants before the intervention begins. If participants lack a home computer/tablet or internet, we loan them a Wi-Fi- enabled tablet and keyboard for use during the study, a process used successfully in another trial in advanced cancer [25].

#### Education-enhanced usual care control

EUC participants will receive a printed NCI educational book on cancer survivorship [30] to ensure uniform access to high-quality symptom management and other information across study arms. To ensure similar access to this information in both conditions, this book is sent to all participants, including those in the *REVITALIZE* arm. In addition, EUC participants will have ongoing access to supportive care resources (e.g., social work, psychology, chaplains) available to patients at treating clinics.

Following our published recommendations [39], to account for potential differences in supportive care resources by site, participants in both study arms will report their use of supportive care services used outside of the study (e.g., non-study supportive care use) with a tracker we piloted in a recent trial [36]. We will account for use of non-study supportive care in analyses of outcomes.

#### Intervention training, facilitation, and fidelity

Similar to the *REVITALIZE* pilot, University of Colorado Boulder clinical psychology doctoral students and post-docs who have completed at least one year of training in behavioral therapy will serve as the *REVITALIZE* interventionists. MPIs Arch and Wright provide training in *REVITALIZE* manual content and the ACT approach using experiential role-playing and coaching, evidence-based training approaches [40, 41] used in the pilot and Arch’s other ACT studies [36, 42].

Weekly supervision is provided, and intervention sessions are videotaped for supervision purposes. After each session, study interventionists report on their adherence to the protocolized content for that session using a checklist. Finally, 20% of *REVITALIZE* sessions will be randomly selected for content fidelity ratings by ACT-trained doctoral students or post-docs who are not involved in managing the study.

### Study ethics and integrity

The Dana-Farber Cancer Institute (DFCI) serves as the single IRB for the study. The internal Data Safety and Monitoring Board at DFCI provides additional oversight for data quality, participant safety, and assessment of potential harms. Verbal informed consent is obtained from all study participants, preceded by a verbal discussion of the consent form in person or by phone with a trained study RA to facilitate full understanding prior to signing. At the beginning of the baseline survey in REDCap, participants sign a HIPAA release appropriate to their site to grant permission for the study team to confirm medical eligibility and treatment information in their medical records. Following completion of the baseline survey, the participant is then sent a wireless electronic pillbox (Wisepill) and completes an orientation with a trained study RA, so the participant understands how to use the device. Study coordinators randomize participants using a sequence created by the study biostatistician. The sequence is uploaded into REDCap using the embedded randomization function wherein the sequence is hidden from the study team to reduce potential bias and once assigned cannot be altered. Data confidentiality is ensured by the collection and storage of patient-reported outcome data in REDCap, a HIPAA-compliant system. To reduce potential bias in outcome assessment, REDCap survey links are sent to participants directly. Participant retention and timely data collection are promoted by compensating participants for their time in completing the online assessments. All research team members complete relevant Collaborative Institutional Training Initiative (CITI) training and all university-affiliated members report an annual Disclosure of External Professional Activities (DEPA).

Each study recruitment site completes a site initiation visit prior to onboarding in which study details are presented and discussed. The MPIs have a standing weekly to biweekly meeting and the study coordinators have a standing weekly to biweekly meeting with the MPIs to ensure timely study communication, coordination, and troubleshooting across the administrative sites. Protocol modifications will be submitted for approval to the DFCI IRB and once approved, communicated to the study coordinators at this standing meeting. The senior leadership team (e.g., study Co-Is) meets regularly to review recruitment rates, protocol changes, and study-related updates.

### Patient advisory committee

The study is guided by a patient advisory committee consisting of up to five diverse patients with advanced ovarian cancer who meet quarterly with the study PIs. In the first year, the advisory committee reviewed study materials, surveys, and recruitment strategies to ensure that they were both culturally sensitive and minimally burdensome. In subsequent years, the advisory committee will meet quarterly to review participant recruitment and retention, help with necessary problem-solving throughout the trial, and provide input on study findings, and strategies and networks to disseminate findings.

### Study timeline

As described in *Study Design*, patient-reported primary and secondary outcomes and process variables are assessed at five timepoints: baseline (pre-intervention), mid-intervention (8 weeks after baseline), post-intervention (13 weeks after baseline), and 20- and 28-week follow-up (FU) with 20-week FU serving as the main endpoint and 28-week FU serving to test maintenance or change in effects over a longer time period. After consenting to participate in the study, steps are done in the following sequence, with the next step initiated after the previous one is completed: (1) participants complete the baseline survey; (2) participants begin using the Wisepill wireless adherence tracking devices [33] for 2 weeks to establish a baseline for PARP inhibitor adherence; (3) participants are randomized to condition. Participants will then continue with Wisepill use, their assigned condition, and the study assessments for nearly 7 months.

### Measures

For their time spent completing the surveys in REDCap, as well as to promote participant retention and on-time data collection, participants will receive $20 per survey assessment plus $5 bonus for completion within 48 h of receiving the survey link, for a total of up to $25 per assessment point. The study enrollment, intervention, and assessment timepoints are displayed in Table 1. If participants choose to drop out of the intervention, or develop disease progression during the study but wish to remain in the study, they will retain the option of completing the assessments. In addition, we will compensate participants $25 for returning their supportive care tracking sheet and Wisepill device, for a total of up to $150 per participant across the study.

#### Primary outcome (Aim 1)

The primary outcome of fatigue interference will be assessed using the well-validated 7-item *Fatigue Symptom Inventory (FSI) Fatigue Interference subscale* [28, 29]. Items rated on an 11-point scale (0 = no interference; 10 = extreme interference) assess the extent to which fatigue in the past week interfered with general activity levels, bathing and dressing, work activity (including housework), concentration, relationships, life enjoyment, and mood. This scale has good internal consistency in cancer populations (α = 0.94-0.95) [29] as well as good convergent and discriminant validity and sensitivity to change following intervention [28].

#### Secondary outcomes (Aim 1)

Patient-reported secondary outcomes were tested in the *REVITALIZE* pilot study [27] and reflect measures selected for psychometric strength and brevity, to reduce patient burden. The *PROMIS Cancer Fatigue Short Form*, 7-item measure [43] evaluates fatigue severity [43, 44]. The *Fatigue Self-Efficacy Scale* [45, 46] evaluates people’s belief in their ability to improve their fatigue. The *Fatigue Catastrophizing Scale* [47, 48] assesses catastrophizing about fatigue, and the *Quality of Life*,* Functional Assessment of Cancer Therapy-Ovarian* [49–51] assesses physical, emotional, social/family, and functional well-being.

#### PARP inhibitor adherence assessment (Aim 2)

To assess monthly PARP inhibitor adherence and non-persistence, we will use Wisepill, a simple wireless electronic pillbox monitoring device (4.3”x1.8”x.5”) that sends real-time data to a protected online server when opened. This enables quick identification of any device issues and transmitting full data even if devices are not returned. Wisepill is highly reliable, acceptable, and easy to use [52–55]. It shows similar reliability as MEMS caps [56], greater accuracy than real-time reporting [57], pill counts or self-report [35, 56, 58], and direct association with biologically-measured adherence markers [57]. We used Wisepill to assess anti-cancer medication adherence in prior work [35, 52, 59]. This work established successful procedures for the remote delivery, instructions, and return of Wisepill monitors using post mail that we use in the current study. Study RA’s also complete a phone orientation with each participant to instruct them on how to use the Wisepill and confirm that it is working properly.

Wisepill adherence tracking device data will compute monthly PARP inhibitor adherence as the percent of days per 30-day month that the tracking device is opened once for each dose prescribed that day, divided by 30, using recommended methods for Wisepill data preparation and analysis [53]. PARP inhibitors that are taken once daily will require one Wisepill opening to be considered adherent on that day, whereas PARP inhibitors taken once in AM and once in PM will require two Wisepill openings at separate times to be considered adherent on that day. (As noted above, we stratify by PARP inhibitor regimen to take into account dosing schedules across study arms).

Wisepill-assessed monthly adherence will serve as the main adherence outcome. Wisepill, along with medical chart review, will also assess PARP inhibitor non-persistence, measured as stopping the PARP and not restarting through study end. Finally, medical chart review will be used to evaluate PARP inhibitor dose reductions and interruptions, the latter defined as breaks in PARP inhibitor use that reflect stopping and starting again during the study rather than stopping through study end, and disease progression events.

#### Intervention processes and mechanisms (Aim 3)

To test theory-based, therapeutic ACT processes and mediators in *REVITALIZE*, we use the *Experiences Questionnaire-Decentering* scale [60] to assess cognitive defusion and acceptance, and the *Valuing Questionnaire* [61] to assess aligning behavior with personal values.

### Data analytic approach

The statistical analysis will be conducted according to the intention-to-treat principle, with all randomized participants included in the primary analyses. Descriptive statistics will be used to evaluate baseline and follow-up outcome measures, including assessment of distributions, potential outliers, and patterns of missing data. For continuous outcomes, we will summarize mean changes from baseline with corresponding 95% confidence intervals (CIs) at each follow-up time point for both groups (Enhanced Usual Care vs. *REVITALIZE*). For binary outcomes (e.g., presence of PARP inhibitor dose delays, interruptions, or discontinuation), we will estimate proportions with corresponding 95% CIs. If clinically meaningful imbalances are observed between groups, adjusted analyses using regression or stratified models will be performed.

For Aim 1, the primary analysis of change in fatigue interference at week 20 will tested using a a two-sample t test (without covariate adjustment). As a secondary analysis, linear regression (ANCOVA) of the outcome on the intervention indicator, adjusting for the baseline value of the outcome and prespecified baseline variables (race/ethnicity, education, age, and income) will be used. If any baseline variable shows imbalance (standardized mean difference > 0.10), it will be retained in the regression model; otherwise only the baseline value of the outcome will be included alongside the variable for condition. Longitudinal mixed-effects models will be conducted as additional secondary and sensitivity analyses. Aim 2 adherence outcomes will be assessed using mixed-effects models for Wisepill data and logistic regression for binary adherence indicators. Aim 3 will evaluate whether the conditions differ on change over time in the hypothesized process variables for *REVITALIZE—* acceptance and values alignment. In addition, we will test mediation of intervention effects on fatigue interference through acceptance and values alignment using the linear regression mediation framework outlined below as well as the multiple mediation framework of Preacher and Hayes [62], with sensitivity analyses using structural equation models.

Data transformations or non-parametric approaches will be considered if model assumptions are not satisfied. Sensitivity analyses will be conducted in the per-protocol population, and all results will be reported in accordance with CONSORT guidelines. Regarding missing data, we will compare baseline characteristics of patients with complete follow-up to those without by randomized condition to assess potential biases in the complete case analysis. We will conduct sensitivity analyses using all available case analysis (among the subset of participants with complete data at baseline and one post-baseline point) as well as additional methods such as multiple imputations by chained equations.

### Power and sample size estimation

We plan to enroll a total of 240 patients (approximately 120 per condition) in the study. Based on findings from the *REVITALIZE* pilot study and our recent RCT for adults with advanced cancer [25], we anticipate 20% missing data at 20-week FU. Accordingly, we expect data from 192 patients (*n* = 96 per group) to be available for the primary analysis of the primary endpoints for each Aim.

For Aim 1, the primary analysis will use a two-sample t-test. With a sample size of 192, the study will have 80% power to detect a significant intervention effect corresponding to Cohen’s d = 0.41 (a small-to-moderate effect size) at a two-sided alpha level of 0.05, and 94% power to detect a moderate effect size (d = 0.50). These thresholds are smaller than the effect sizes observed in the pilot trial [27], where the primary endpoint yielded approximately an effect size of d = 0.94 for the *REVITALIZE* intervention group versus EUC at follow-up, and the targeted secondary endpoints yielded effect sizes of d≥0.60. Thus, the planned sample size provides sufficient power to detect clinically meaningful effects for Aim 1.

For Aim 2, the primary analysis for chart review data will use Fisher’s exact test. With a sample size of 192, the study will have 80% power to detect an intervention effect corresponding to Cohen’s h = 0.4 (a small-to-medium effect size) at a two-sided alpha level of 0.05, and 93% power to detect a moderate effect size (h = 0.5). The longitudinal outcomes, assessed using monthly Wisepill adherence data, provide even greater statistical power than the chart review outcomes because they involve repeated continuous (rather than single binary) measurements. Accordingly, we consider the study to be adequately powered for the outcomes specified in Aim 2 [63].

For Aim 3, the power and sample size considerations are based on mediation analysis using linear regression models [64]. Let $\:Y$ denote the change in the primary outcome from baseline to 20 week FU, $\:Z$ the intervention indicator, and $\:M$ the mediator. We consider two models, Model 1: $\:E\left(Y|Z\right)={\beta\:}_{0}^{*}+{\beta\:}_{1}^{*}Z$; and Model 2: $\:E\left(Y|Z\right)={\beta\:}_{0}+{\beta\:}_{1}Z+{\beta\:}_{2}M$. We define $\:({\beta\:}_{1}^{*}-{\beta\:}_{1})/{\beta\:}_{1}^{*}$ as the proportion of treatment effect explained (PTE) and test the null hypothesis of no mediation effect (i.e., PTE = 0). Power at a two-sided 0.05 alpha for detecting mediation depends on the mediator effect size, the residual error standard deviation (SD), and the correlation between the intervention and mediator. Based on pilot data [27, 65], we conservatively estimate the SD of the residual error for the primary endpoint to be approximately 1.8. Under a correlation between condition and mediator ranging from 0.1 to 0.5, the study with *n* = 192 achieves at least 84% power to detect a mediator with the effect size of d≥0.4.

### Dissemination of study results and data sharing

Once the trial is complete, we plan to publish our findings in peer-reviewed journals, present our findings at scientific meetings, and ensure that our study results are submitted to clinicaltrials.gov within 12 months of study completion. In addition to dissemination to academic audiences, we will also present our study results to patient advocacy organizations and share study results with interested participants with a plain language summary. After the primary endpoints are published, we also plan to share de-identified participant data, relevant statistical code, and other study materials with interested researchers upon submission of a formal request for data sharing.

## Discussion

Among adults with advanced ovarian cancer reporting moderate to severe fatigue while on PARP inhibitors, this two-arm RCT will evaluate whether *REVITALIZE* reduces fatigue interference and related outcomes and improves PARP adherence and quality of life more than an education-enhanced usual care control condition. As fatigue is a common side effect of many emerging anti-cancer treatments [4], this study will inform whether behavioral interventions can reduce fatigue-related effects of newer treatments in advanced cancer more broadly.

If successful, this study will make the following contributions: (1) By adequately powering the study, we will determine whether *REVITALIZE* improves fatigue interference and broader fatigue outcomes for patients with advanced ovarian cancer experiencing fatigue on PARP inhibitors, thereby advancing the limited evidence base regarding interventions for fatigue in advanced cancer [4]; (2) By examining whether reducing fatigue interference leads to better PARP inhibitor adherence, this study addresses a largely overlooked question of whether reducing fatigue side effects can improve adherence to anti-cancer treatment—which would inform clinical care guidelines; (3) By conducting the intervention entirely remotely and online for patients in multiple states, this study evaluates an intervention with more reach and scalability potential than interventions that rely on in-person, local delivery; (4) By recruiting participants from both academic and community clinics, this study will test *REVITALIZE’s* efficacy among patients treated within a broad range of contexts in which patients receive care; (5) By evaluating the intervention’s processes of therapeutic change, this study will inform how *REVITALIZE* works.

In summary, *REVITALIZE* has the potential to advance the science and care for patients living with advanced cancers who face life-interfering fatigue – an understudied and growing population with profound unmet supportive care needs. By rigorously evaluating *REVITALIZE* in a well-powered RCT, this study will provide key evidence for a remotely-delivered intervention that can be adapted to improve supportive care in other advanced cancers.

## Supplementary Information

Supplementary Material 1.

## Data Availability

No datasets were generated or analysed during the current study.
